# Study of the relationships between brachytherapy plan parameters in an attempt to verify total dwell time in vaginal cylinder applications

**DOI:** 10.1002/acm2.12341

**Published:** 2018-05-13

**Authors:** Emmanuel O. Oyekunle, Bidemi I. Akinlade, Mutiu A. Jimoh

**Affiliations:** ^1^ Department of Radiation Oncology University College Hospital Ibadan Nigeria; ^2^ Department of Radiation Oncology College of Medicine University College Hospital Ibadan Nigeria

**Keywords:** brachytherapy, parameters, total dwell time, vaginal cylinder

## Abstract

**Purpose:**

Source strength (S_k_), sizes of vaginal cylinder applicators (VCA), number of dwell positions (DPs), and the prescribed dose (D) are basic parameters in brachytherapy (BT) treatment planning contributing to total dwell time (TDT). This study was aimed at assessing the relationships between the specified variables in an attempt to verify the TDT in high‐dose‐rate (HDR) vaginal cylinder applications.

**Methods:**

One hundred and twenty‐one patients treated with Gynesource‐Co^60^ (Bebig, Germany) using VCAs of diameters 20, 25, and 30 mm at University College Hospital, Nigeria, were enrolled in this study. Brachytherapy doses ranging from 3 to 7 Gy were always prescribed to points 5 mm away from the cylinder's surface. Treatment planning was undertaken on HDR‐Basic treatment planning system (TPS) which utilizes source step size of 5 mm. Data on the stated parameters related to the first BT fractions of the patients were acquired. With the aid of EViews statistical software, two forms of mathematical models were thereafter developed. The resulting TDTs from the models were compared with the TPS values using Minitab statistical software.

**Results:**

The relationships obtained for the increasing sizes of the VCA were $TDT_{1}\left(min\right)=2.22+3.17\left(\frac{D}{S_{k}}\right);TDT_{1}\left(min\right)=3.52+3.74\left(\frac{D}{S_{k}}\right);TDT_{1}\left(min\right)=-1.96+6.91\left(\frac{D}{S_{k}}\right)\;and\;TDT_{2}\left(min\right)=0.50-0.03S_{k}+0.02D+0.55DP_{s};\;TDT_{2}\left(min\right)\;=\;7.08\;-\;0.06S_{k}\;+0.02D+0.67DP_{s};TDT_{2}\left(min\right)=7.02-0.11S_{k}+0.03D+1.25DP_{s}$ The model‐based TDTs correlate with the TPS‐calculated values with r_1_ = 0.80 (*P* = 0.412) and r_2_ = 0.97 (*P* = 0.468).

**Conclusions:**

The findings of this study could suggest likely variations in the treatment time when certain changes occur in the related parameters. The increasing size of the vaginal cylinder has a positive influence on the brachytherapy treatment time. The latter model has been a useful tool in the verification of the dose delivery time at the first HDR brachytherapy center in Nigeria and West Africa.

## 1 INTRODUCTION

Brachytherapy (BT) is a form of radiotherapy which can be administered as a monotherapy of radiation or in supplement to external beam radiotherapy (EBRT). A peculiar indication for brachytherapy for decades has been gynecological malignancy. The classification of brachytherapy according to rates of dose delivery includes Low‐Dose rates (LDR), Medium‐Dose Rates (MDR), and High‐Dose‐Rate (HDR) techniques.^1^ High‐dose‐rate brachytherapy has, however, become the major practice across the world, particularly in the developed nations.^2^ Its choice as a substitute for the LDR technique is due to the related advantages. These include shortened treatment time, outpatient‐based treatment, and improved patient comfort. In addition, the use of the HDR vaginal cylinders is simple and offers better placement of the applicators in relation to the desired anatomic structures than the tandem‐ring and tandem‐ovoid combinations. There is also a better dosimetric control for the small and high‐intensity source that moves through the cylinder at 5‐mm intervals.^3^ The HDR technique, however, requires a stricter level of quality assurance (QA) to ensure adequate safety in its operations including the computerized treatment planning. Each step in the integrated process of RT needs quality control and quality assurance to prevent a number of errors and to give high confidence that patients will receive the prescribed treatment correctly.^4^, ^5^, ^6^

One vital component of the QA is the independent checks of total dwell times for brachytherapy dose delivery by manual calculations or a second computer program. The success of brachytherapy in managing gynecological cancers can be defeated when treatment misadministration occasioned by the use of wrong treatment times occur. If the dose delivery time is less than required, there would be an underdosing of the target volume thereby increasing the likelihood for tumor recurrence. If the reverse is the case, the related normal organs‐at‐risk (OAR) such as the bladder and the rectum may experience morbidity depending on their level of overexposure. The potential risk to the OARs is higher in the HDR technique that offers a relatively larger fractional dose in a short period of minutes. Air‐kerma strength (S_k_), diameters of vaginal cylinder applicators VCA), treatment length (L), or number of dwell positions (DPs) and the prescribed dose (D) are basic parameters in brachytherapy treatment planning contributing to the total dwell time (TDT). This study was therefore aimed at investigating the relationships between the specified variables in an attempt to verify the dose delivery time in HDR vaginal cylinder applications.

## 2 MATERIAL AND METHODS

**Figure 1:**
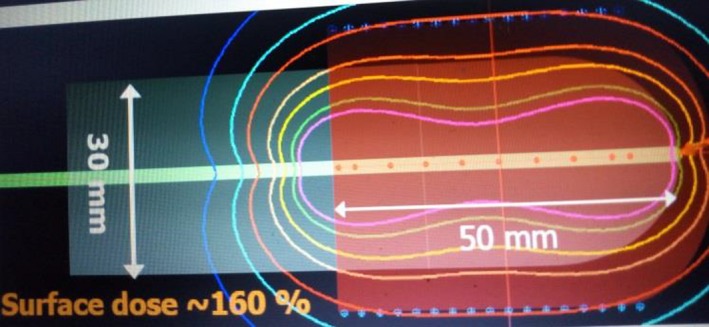
An illustration of a typical brachytherapy treatment plan for 30 mm diameter (grey area) vaginal cylinder. Dose (red isodose) prescription was only to points 5 mm away from the surface of the cylinder for a treatment length, 5 cm (pink area) along the applicator in this case. (For a clearer illustration, this figure was culled and modified (with permission) from a lecture presentation of Tara Hellebust entitled ‘Physics aspects of treatment planning in endometrium cancer’.)

Between July 2008 and August 2013, HDR brachytherapy was delivered to over 450 patients who presented with gynecological cancers at the Department of Radiation Oncology, University College Hospital (UCH), Ibadan, Nigeria. The volume of patients given intracavitary brachytherapy (ICBT) within the specified period at UCH, the pioneer academic tertiary health institution in Nigeria, could have been exceeded if there was no equipment downtime. The use of VCA accounts for about 30% of intracavitary applications at UCH. The procedural application of VCA at our institution had been described previously.^7^ A total of 121 patients who had HDR BT within the 5‐year period were enrolled in this retrospective study. They were treated with Gynesource afterloader (Bebig, Germany) with a Cobalt‐60 source using VCAs of diameters 20, 25, and 30 mm. Patients’ characteristics including the distribution of the applicator sizes among them within the study period are presented in Table 1. Dose prescription for treatment plans (TPs) involving a single catheter at UCH, Nigeria, is often to points 5 mm away from the surface of the cylinder only as illustrated in Fig. 1. The number of dwell positions applicable to a TP would normally increase with the length of treatment required along the vaginal cylinder. The HDR‐Basic TPS (Bebig, Germany) associated with the brachytherapy equipment employs the use of source step size of 5 mm only. Therefore, treatment lengths at the institution which normally range from 2 to 10 cm correspond to source activation at 4–20 DPs along the catheter.

**Table 1:** Patients’ Characteristics (*n* = 121) and distribution of vaginal cylinder applicators within a 5‐year period

Features	Frequency	%
Age (years)		
Range	27–69	
Mean	47	
FIGO stage		
IB	9	7.44
IIA	18	14.88
IIB	29	23.97
IIIA	42	34.70
IIIB	7	5.79
IVA	16	13.22
VCA diameter		
20 mm	36	29.75
25 mm	53	43.80
30 mm	32	26.45

FIGO, International Federation of Gynecology and Obstetrics.

Mayo and Ulin^8^ earlier described a method for checking the treatment time calculation for HDR vaginal cylinder treatments. For dose prescription points located only at 5 mm away from cylinder surface, the authors proposed the determination of a scaling factor *K* that relates the prescribed dose *D*, the source strength *Sk*, and the total treatment time *TT*, in the form of

(1) TT=K×D/SK

In this study, we acquired dosimetric data on Cobalt‐60 air‐kerma strength, doses to the prescription points, numbers of dwell positions, and the TDT related to the initial ICBT applications of the subjects considered. The first and the second parameters were re‐expressed in cGy.cm^2^/min and cGy, respectively, in order for TDTs to be obtained directly in minutes which is realistic for HDR treatments. EViews (version 9) statistical software (Canada) was used on the three sets of data (based on applicator diameters) to perform a regression analysis in two phases. Firstly, the three quantities highlighted in eq. (1) were modeled to verify their relationship.

Regression analysis of the treatment planning data involving three parameters yielded a relationship given as:

(2) $$\text{TDT}=\beta_{0}+\beta_{1}\left(D/S_{k}\right)$$

This is a resemblance of eq. (1) in literature.

By comparing the resulting eq. (2) with the previous, a mathematical expression (eq. (3)) for the treatment time factor was obtained.

While *β*_0_ is the intercept, *β*_1_ being the slope represents the treatment time factor which can be evaluated as:

(3) $$K=\left(\text{TDT}-\beta_{0}\right)/\left(D/S_{k}\right)$$

The values of the parameter K were therefore evaluated for the three sizes of the VCA. The other stage involved the inclusion of the number of the DPs as an additional parameter in the statistical analysis.

Further regression analysis incorporating the treatment length factor (number of DPs) resulted in a mathematical relationship as follows:

(4) $$\text{TDT}=\beta_{0}+\beta_{1}S_{k}+\beta_{2}D+\beta_{3}\text{DP}$$

The two models developed were used to generate predicted dose delivery times which were compared with the corresponding TPS values.

## 3 RESULTS

The descriptive of brachytherapy plan parameters and the corresponding *P*‐values as determined by the analysis of variance are presented in Table 2.

**Table 2:** Descriptive of treatment plans’ parameters and related *P*‐values

Brachytherapy parameters	VCA 20 mm	VCA 25 mm	VCA 30 mm	ANOVA *P*‐value
I. Sk (cGy.cm^2^/min)				
Min.	161.51	161.62	160.36	
Max.	303.07	308.10	273.31	0.000
Mean	223.59	220.24	186.10	
II. DPs				
Min.	4	4	4	
Max.	20	28	16	0.371
Mean	9.28	10.36	10.16	
III. D (cGy)				
Min.	400	300	400	
Max.	700	700	700	0.023
Mean	569.44	555.660	620.31	
IV. TDT (min)				
Min.	3.45	4.72	9.48	
Max.	17.6	24.20	36.6	0.000
Mean	10.79	13.54	21.70	

The values of the coefficients *β*_0_ and *β*_1_ for the respective diameters of the applicators are given in Table 3. In Table 4, the resulting K values evaluated using eq. (3) are presented.

**Table 3:** Combined results of regression analysis involving three treatment planning variables for the three diameters of the vaginal cylinders (Model 1 Pattern)

VCA	TDT		
	20 mm	25 mm	30 mm
β_0_	2.220 (1.359)	3.516 (1.127)	−1.955 (3.451)
β_1_ (D/S_k_)	3.166 (0.479)	3.742 (0.398)	6.912 (0.983)
R‐sq.	0.562	0.634	0.622
R‐sq(Adj)	0.549	0.627	0.610
Prob(F‐statistic)	0.000	0.000	0.000
S. E. of reg.	2.441	0.044	0.072
Observations	36	53	32

**Table 4:** Treatment time factors, K, evaluated for the three sizes of the vaginal cylinder applicator

20 mm VCA		25 mm VCA			30 mm VCA	
0.71	3.36	0.73	3.64	4.59	3.81	7.53
1.33	3.38	1.83	3.65	4.62	4.61	7.72
1.66	3.39	1.99	3.68	4.77	4.92	7.85
1.73	3.4	2	3.73	4.85	5.24	8.08
2.08	3.4	2.37	3.74	4.89	5.38	8.50
2.36	3.4	2.47	3.8	5.78	5.52	8.74
2.36	3.51	2.6	3.81	5.86	6.26	8.99
2.51	3.51	2.65	3.86	5.92	6.27	9.03
2.61	3.6	2.71	3.86		6.81	9.10
2.75	3.75	2.83	3.87		6.86	
2.79	4.0	2.96	4.07		6.87	
2.83	4.18	3.02	4.07		6.88	
2.88	5.07	3.07	4.11		6.89	
3	5.59	3.21	4.15		6.92	
3.03	6.05	3.27	4.17		6.92	
3.03		3.28	4.23		6.93	
3.05		3.29	4.24		6.95	
3.08		3.47	4.28		6.98	
3.21		3.49	4.33		6.98	
3.25		3.49	4.34		6.98	
3.32		3.51	4.35		7.00	
		3.64	4.36		7.04	
			4.47		7.35	

The intercepts and the coefficients of the variables related to eq. (4) for the three cylinder sizes are therefore presented in Table 5.

**Table 5:** Combined results of regression analysis involving four treatment planning variables for the three diameters of the vaginal cylinders (Model 2 Pattern)

VCA	TDT		
	20 mm	25 mm	30 mm
β_0_	0.497 (2.600)	7.085 (2.003)	7.015 (4.834)
β_1_ (S_k_)	−0.033 (0.006)	−0.060 (0.006)	−0.106 (0.016)
β _2_ (D)	0.022 (0.003)	0.023 (0.002)	0.035 (0.004)
β _3_ (DPs)	0.553 (0.082)	0.666 (0.058)	1.255 (0.111)
R‐sq.	0.847	0.897	0.930
R‐sq(Adj)	0.832	0.891	0.923
Prob(F‐statistic)	0.000	0.000	0.000
S. E. of reg.	1.489	1.431	1.930
Observations	36	53	32

Regression analyses in both cases were at a level of significance of 0.01.

**Figure 2:**
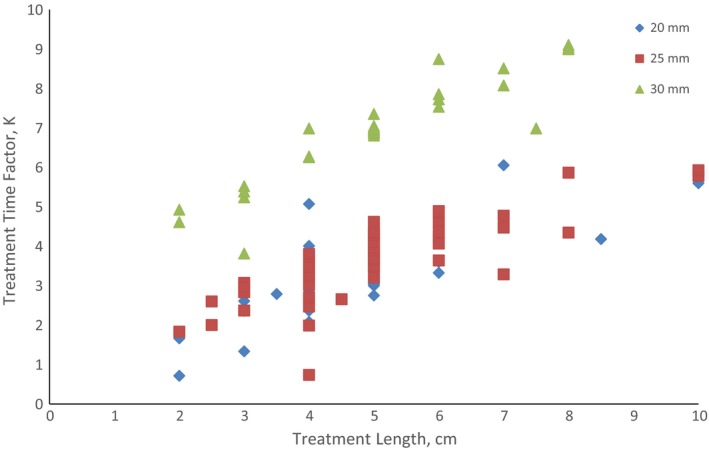
Variation of treatment time factors, K, with treatment lengths on the three diameters of the cylinder applicator for doses prescribed to a depth of 5 mm.

**Figure 3:**
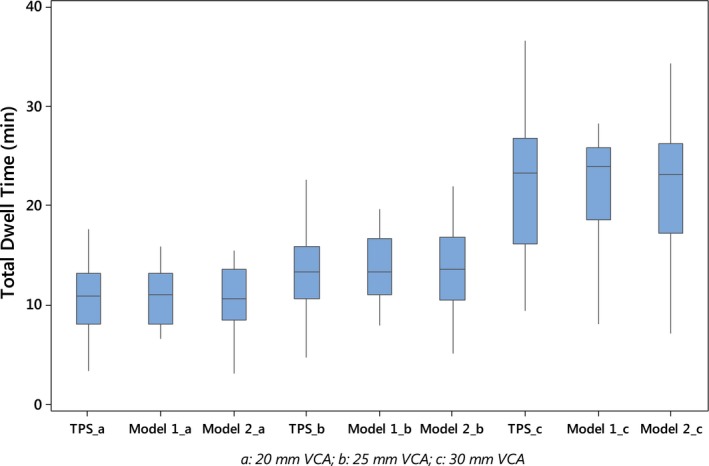
Boxplots comparing TPS‐calculated total dwell times with predictive values resulting from the models for the three diameters of the cylinder applicator.

In Fig. 2, the variation of the treatment time factors, K, with the related number of dwell positions along the applicator is illustrated for the respective sizes of the cylinder. Figure 3 shows boxplots comparing total dwell times calculated by the TPS with the corresponding values predicted by the two model patterns for cylinders 20, 25, and 30 mm, respectively.

The model‐based TDTs correlate with the TPS‐calculated values with r_1_ = 0.80 (*P* = 0.412) and r_2_ = 0.97 (*P* = 0.468). Comparing the predictive treatment times of both patterns with each other, we obtained r = 0.814, with no statistical significant difference (*P* = 0.394).

## 4 DISCUSSION

Cancer involving the vagina may be primary, recurrent, and occasionally, metastatic. The vaginal apex is the most common site of local recurrence of endometrial cancer following hysterectomy.^9^ Brachytherapy applications to the vaginal vault using cylinder applicators at UCH, Nigeria, are often administered to patients with or without hysterectomy having histological diagnosis of vaginal, cervical, and endometrial cancer. Patients without hysterectomy rather had a bulky tumor that necessitated the use of the VCA. Authors across Africa have confirmed that a majority of the patients in their studies had late FIGO stage presentations.^10^, ^11^, ^12^ The situation is the same at our institution following the trend obtained in Table 1 of this study. The diameter of an applicator, treatment length, prescribed dose, and the source strength at the time of a brachytherapy application are parameters fundamental to the determination of the treatment duration following the implant procedure. The descriptive statistics of brachytherapy plan parameters are presented in Table 1. The minimum values of the air‐kerma strength across the three applicator sizes are quite comparable. The maximum S_k_ related to the 30 mm VCA which was lower than for other patient groups only indicated that some patients were treated with the applicator on later dates. Treatment lengths along the applicator within the study duration ranged from 2 to 10 cm (4–20 DPs). While the prescribed doses and number of DPs are comparable in the three study groups, an increasing trend of TDTs with applicator sizes was obtained. This could be due to variation in S_k_ and more importantly the diameter of the cylinder. As stated in the source certificate, the initial air‐kerma strength of the HDR Cobalt‐60 (Bebig, Germany) at UCH was 22870 cGy.cm^2^ h^−1^ [381.17 cGy.cm^2^ min^−1^] corresponding to the typical activity of 74.74 Gy available commercially. Overall, the S_k_ ranged from 9,621.6 to 18,486 cGy.cm^2^ h^−1^ [160.36–308.10 cGycm^2^ min^−1^] within the study period of 5 years.

Results of ANOVA (Table 2) show there are no statistically significant differences in the number of dwell positions (*P* = 0.371) and the prescribed doses per fraction (*P* = 0.023). This is very obvious as the range of DPs and doses used in treatment planning was very similar across the VCA sizes. However, the means of S_k_ and TDTs related to the various applicators differ significantly (*P* = 0.000). This should be the case as different applicators were used for ICBT at varying times, therefore necessitating appreciable differences in S_k_ at the treatment dates. Consequently, the dose delivery time would differ considerably considering the feasible changes in source strength and the varying dimensions of the intravaginal applicator.

Table 3 presents the combined outputs of the regression analysis involving three variables. We recall that a treatment time factor, K, was earlier identified in the study of Mayo and Ulin.^8^ According to the authors, the cylinder radius, prescription radius, and the active lengths prescribed to 5 mm depth and to the surface of the VCA were both given considerations in the determination of K. This study considered the sizes of the VCA separately in the process of evaluating K for dose prescriptions to 5 mm away from the cylinder's surface only. The coefficient *β*_1_ in Table 3 which is representative of K showed a significant dependence on the cylinder radius which normally determines the prescription radius for a given treatment plan.

It is therefore a precursor to the pattern of the evaluated treatment time factors, K, presented in Table 4, showing variability with treatment lengths along the different applicator sizes as illustrated in Fig. 2.

In general, it was found to have increased with the number of dwell positions activated for treatment. Moreover, the factor becomes more significant as the applicator becomes bigger in size. This could be attributed to the fact that the proximity of the dose prescription points to the source positions at the center of the intravaginal applicator (Fig. 1) will vary with the cylinder size. To this effect, the TDT to deliver a given dose would be influenced accordingly. The above trends are comparable to the outcomes of the study of Mayo and Ulin involving same sizes of the VCA.^8^ However, the values of K obtained in this work are generally relatively higher which could be mainly attributed to significantly lower S_k_ associated with the HDR Cobalt‐60 source in comparison to Ir‐192. This warranted longer treatment times (Table 2) obtained in this study. The dose delivery time for a HDR plan with a Co‐60 would be 1.8 times longer than that with Ir‐192 when both sources have their initial S_k_ of 22,645 and 40,820 cGy·cm^2^ h^−1^ [377.417 and 680.333 cGy·cm^2^ min^−1^], respectively.^13^ While Iridium‐192 is the primary radionuclide used for HDR brachytherapy, the choice of Co‐60 at our institution is premised on economic factor. An average of 20 source replacements for Ir‐192 is needed for a corresponding single‐source replacement of a Co‐60 source. An iridium source replacement would require an average cost of about 15,000 USD, culminating in a total of about 300,000 USD for a 60‐month period. However, the cost of a Co‐60 source change during the same period (after its half‐life of 5 years) is about 34,000 USD.^14^

Table 5 presenting a *P*‐value of 0.000 shows the combined relevance of S_k_, dose, and DPs in the evaluation of treatment time. The R^2^ values obtained in this case imply that source strength, dose, and number of dwell positions jointly account for 83.2%, 89.1%, and 92.3% variation in total dwell times for 20, 25, and 30 mm vaginal cylinder applications, respectively. As such, 16.8%, 10.9%, and 7.7% variations in the treatment time are being explained by other factors outside the models. This study tells the relationship of each of the brachytherapy parameters with the total treatment time for the different applicator sizes. For a VCA of 20 mm, an additional 1 cGy in the prescription dose to 5 mm from the cylinder surface will yield an increased TDT by 0.022 min when the source strength and number of dwell positions remain unchanged. This value becomes 0.023 and 0.035 min for the larger applicators. An increase of 1 Gy in D will bring about time elongation by 2.2, 2.3, and 3.5 min. In teletherapy or brachytherapy, it is in order for treatment time to increase with the dose. This study indicated that the degree of increase in the TDT is highest with the use of the 30 mm applicator. This could subject the OAR to a relatively higher dose. This inference is drawn from a previous study 7 at our institution where the maximum rectal TLD doses were associated with the use of 30 mm VCA for treatment lengths of 5–8 cm. The in‐vivo dose effect of applicator size was, however, reduced when the treatment length was <4 cm. Similarly, the number of DPs depicting the length of treatment along the vaginal applicator varies positively with TDT. According to the results of our study, the inclusion of an additional dwell position in brachytherapy planning with the 20 mm VCA will increase the TDT by result in 0.553 min. This positive time change increases to 0.666 and 1.255 min in the case of the 25 mm and the 30 mm applicators. It thus means that an extension of the treatment length by 1 cm (corresponding to two additional DPs) along the VCA would engender a longer TDT by twice the above time durations. The *P*‐value of 0.000 in all cases is statistically significant at 1% significance level. For a given source strength and prescribed dose, a 1 cm increase in treatment length will engender elongation of the dose delivery time by 1.11, 1.33, and 2.51 min for 20, 25, and 30 mm VCAs, respectively. According to our knowledge, this is the first study examining the relationships between the three BT parameters and the treatment time with respect to varying applicator diameters in ^60^Co cylinder applications. There is therefore no published data with which findings in certain aspects of this study could be compared. In the boxplots for the 20 mm VCA shown in Fig. 3, the whisker of the TPS‐calculated treatment times extends within the range 3.45–17.6 min. Corresponding predictive values by the models are 6.61–15.94 min and 3.18–15.51 min sequentially. Therefore, the whiskers of the predicted values arising from model set 1 and 2 covers 65.9% and 89.1% of the actual TDT range, respectively. The trend is same for other applicator sizes. Corresponding proportions with respect to the 30 mm cylinder were 74% and 99.8% of the TPS‐calculated treatment times. Figure 3 which illustrates the patterns of the dose delivery times and Tables 6(a)–6(c) presenting cross‐sections of these values affirm that the latter model set is in better agreement with the TPS‐calculated values. The succeeding model has therefore been of use in cross‐checking the dose delivery time for cylinder applications at the pioneer HDR centre in Nigeria. The verification program which requires an input of the S_k_ and the related parameters is spreadsheet based and can be executed in less than a minute. Our institutional study has also helped to gain insights on how the total dwell time for cylinder applications would vary with the treatment parameters in order to be properly guided in HDR brachytherapy administration. This knowledge is also needful when certain changes are made to BT parameters from one treatment fraction to another. As most gynecological cancers present late in Nigeria, there is a great strain on the country's radiotherapy facilities.^10^ A judicious use of the HDR brachytherapy equipment which is grossly inadequate in Nigeria is therefore of the essence.

**Table 6:** A cross‐section of TPS‐calculated treatment times (min.) and model‐predicted values: (a) 20 mm VCA; (b) 25 mm VCA; (c) 30 mm VCA

TPS	MODEL 1	MODEL 2	% diff. (TPS/Model 1)	% diff. (TPS/Model 2)
(a) 20 mm VCA				
7.17	7.44	7.21	3.63	0.55
8.38	8.65	8.81	3.12	4.88
8.55	8.8	8.96	2.84	4.58
9.77	8.85	10.05	−10.40	2.79
14.88	13.63	15.06	−9.17	1.20
11.27	12.15	11.72	7.24	3.84
10.72	10.5	10.36	−2.10	−3.47
12.9	12.17	12.76	−6.00	−1.10
8.57	10.74	8.46	20.20	−1.30
15.57	14.25	15.34	−9.26	−1.50
14.33	14.86	14.56	3.57	1.58
15.57	15.94	14.95	2.32	−4.15
15.6	14.67	15.51	−6.34	−0.58
10.6	10.5	10.36	−0.95	−2.32
12.58	11.99	12.66	−4.92	0.63
(b) 25 mm VCA				
12.92	9.6	13.41	−34.58	3.65
13.3	9.7	13.72	−37.11	3.06
12.45	10.99	12.87	−13.28	3.26
16.4	14.01	16.19	−17.06	−1.30
12.18	13.35	12.52	8.76	2.72
10.57	11.09	10.41	4.69	−1.54
11.93	11.1	11.76	−7.48	−1.45
10.7	11.21	10.65	4.55	−0.47
11.07	11.28	10.77	1.86	−2.79
13.47	11.32	13.51	−18.99	0.30
8.57	11.49	8.49	25.41	−0.94
13.97	12.48	13.94	−11.94	−0.22
7.88	11.67	8.15	32.48	3.31
10.17	11.76	10.28	13.52	1.07
18.03	16.55	17.78	−8.94	−1.41
(c) 30 mm VCA				
29.7	22.1	30.23	−34.39	1.75
27.68	23.42	28.82	−18.19	3.96
18.77	16.29	19.49	−15.22	3.69
23.87	23.81	24.11	−0.25	1.00
24.02	23.99	24.25	−0.13	0.95
24.22	24.19	24.4	−0.12	0.74
24.67	24.34	24.51	−1.36	−0.65
24.12	24.53	24.65	1.67	2.15
33.63	25.27	32.71	−33.08	−2.81
22.83	25.36	22.72	9.98	−0.48
25.72	25.83	25.55	0.43	−0.67
23.77	26.48	23.46	10.23	−1.32
27.1	27.32	26.49	0.81	−2.30
19.52	19.13	19.67	−2.04	0.76
14.47	19.14	14.66	24.40	1.30

VCA, vaginal cylinder applicator; diff, difference.

## 5 CONCLUSIONS

This study on vaginal cylinder applications is with regards to the use of Cobalt‐60 on HDR‐Basic TPS operating with a single‐source step size of 5 mm. The outcomes could suggest likely variations in the treatment time when certain changes occur in the related parameters. The study has demonstrated that elongation of the dose delivery time increases with the size of the VCA. It is needful to ensure the choice of applicator size and the treatment length along the cylinder is always appropriate for the individual patients in order to justify the TDT that would result. The latter model developed has been a useful tool for the verification of the dose delivery time at the first HDR brachytherapy center in Nigeria. Similar studies should be undertaken by other institutions to assess the weights of the existing relationships among the specified parameters. Such works could consider predetermining the total dwell time, particularly for HDR ^60^Co applications, when the dose is prescribed to points 5 mm away from the VCA's surface only.

## CONFLICT OF INTEREST

The authors report no conflict of interest in this study.
